# Mental health in people with Parkinson’s disease during the COVID-19 pandemic: potential for targeted interventions?

**DOI:** 10.1038/s41531-021-00238-y

**Published:** 2021-10-28

**Authors:** L. J. Dommershuijsen, A. Van der Heide, E. M. Van den Berg, J. A. Labrecque, M. K. Ikram, M. A. Ikram, B. R. Bloem, R. C. Helmich, S. K. L. Darweesh

**Affiliations:** 1 Department of Epidemiology, Erasmus MC University Medical Center, Rotterdam, The Netherlands; 2 Centre of Expertise for Parkinson & Movement Disorders, Department of Neurology, Donders Institute for Brain, Cognition and Behaviour, Radboud University Medical Center, Nijmegen, The Netherlands; 3 Department of Neurology, Erasmus MC University Medical Center, Rotterdam, The Netherlands

**Keywords:** Parkinson's disease, Quality of life, Epidemiology, Parkinson's disease

## Abstract

The COVID-19 pandemic has introduced a myriad of challenges to the social life and care of people with Parkinson’s disease (PD), which could potentially worsen mental health problems. We used baseline data of the PRIME-NL study (*N* = 844) to examine whether the association between COVID-19 stressors and mental health is disproportionately large in specific subgroups of people with PD and to explore effects of hypothetical reductions in COVID-19 stressors on mental health and quality of life. The mean (SD) age of the study population was 70.3 (7.8) years and 321 (38.0%) were women. The linear regression effect estimate of the association of COVID-19 stressors with mental health was most pronounced in women, highly educated people, people with advanced PD and people prone to distancing or seeking social support. Smaller effect estimates were found in people scoring high on confrontive coping or planful problem solving. The parametric G-formula method was used to calculate the effects of hypothetical interventions on COVID-19 stressors. An intervention reducing stressors with 50% in people with above median MDS-UPDRS-II decreased the Beck Depression Inventory in this group from 14.7 to 10.6, the State-Trait Anxiety Inventory from 81.6 to 73.1 and the Parkinson’s Disease Quality of Life Questionnaire from 35.0 to 24.3. Insights from this cross-sectional study help to inform tailored care interventions to subgroups of people with PD most vulnerable to the impact of COVID-19 on mental health and quality of life.

## Introduction

Depressive and anxiety symptoms are common in people living with Parkinson’s disease (PD)^1,2^ and can substantially worsen quality of life^3^. The COVID-19 pandemic has introduced challenges to both access to care and to the social life of people with PD, which could potentially worsen mental health problems^4–6^.

In order to slow infection rates of the SARS-CoV-2 virus, drastic social distancing measures have been taken^7^. These disruptions in normal life have caused considerable psychological stress in community-dwelling individuals^8^. Importantly, people with PD are especially vulnerable to this stress for several reasons^9,10^. First, governmental restrictions have hindered physical exercise, an important complementary treatment strategy for PD^11^, which has led to worsening of symptoms^12,13^. Second, due to the deficient central dopaminergic transmission, people with PD typically have disproportionate difficulties with flexible adaptation to rapid and drastic changes in daily routines^14^, such as those introduced by the COVID-19 pandemic^9^.

Several previous studies indeed concluded that the COVID-19 pandemic worsened depressive and anxiety symptoms and reduced quality of life in people with PD^4–6^. However, there is a lack of empirical data on subgroup differences regarding the impact of the COVID-19 pandemic on mental health and quality of life. This lack of insight has so far precluded the deployment of targeted interventions.

The potential improvements in mental health by intervening on COVID-19 stressors is dependent both on the prevalence and effect size of the stressors. By simulating hypothetical interventions on COVID-19 stressors, we can take both factors into account and test the possible effects of interventions targeted at specific subgroups of people with PD. The objective of this study was thus to identify subgroup differences in the association of COVID-19 stressors with mental health in people with PD and to explore whether hypothetical interventions on COVID-19 stressors could improve mental health and quality of life.

## Results

### Population characteristics

Table 1 shows the characteristics of the study population. The mean (SD) age of the participants was 70.3 (7.8) years and 321 (38.0%) participants were women. Most participants lived together with a partner or child (84.4%) and 9.1% had paid employment. Participants were diagnosed with PD at a mean (SD) age of 64.0 (9.1) years. On a scale from 0 to 40, the mean (SD) COVID-19 stressors sum score was 9.6 (5.9). The mean social stressors score (4.9, SD 3.2) was higher than the mean care stressors score (1.9, SD 2.4). The highest scores were found for loss of social contacts (median 2, interquartile range (IQR) 0–3), social events canceled (median 3, IQR 1–4) and unable to perform physical activity or to relax (median 3, IQR 1–4). The mean BDI was 11.6 (6.7), the mean STAI 75.9 (18.6) and the mean PDQ-39 25.8 (13.1). Average cross-sectional BDI, STAI and PDQ-39 scores by date of filling out the questionnaire are shown in Supplementary Fig. 1.

**Table 1:** Baseline characteristics.

*N*	844
Demographics	
Age, years	70.3 (7.8)
Female, *n* (%)	321 (38.0)
Living situation, *n* (%)	
Alone	118 (14.0)
Together	712 (84.4)
Assisted living	14 (1.7)
Education, *n* (%)	
Primary	34 (4.0)
Lower	231 (27.4)
Intermediate	175 (20.7)
Higher	404 (47.9)
Dutch Ethnicity, *n* (%)	836 (99.1)
Work, *n* (%)	
Paid employment	77 (9.1)
Retired, homemaker, volunteer work	636 (75.4)
Incapacity to work	131 (15.5)
Disease related	
Age PD diagnosis, years	64.0 (9.1)
PD duration, years	6.4 (5.4)
MDS-UPDRS-II (0–52)	12.3 (7.5)
SCOPA-AUT (0–69)	17.4 (7.7)
Telephone MoCA (0–22)^a^	17.9 (3.0)
Comorbidities, n (%)^b^	515 (61.0)
Psychiatric comorbidities, n (%)^c^	70 (8.3)
COVID-19 related	
Total COVID-19 influence (0–40)^d^	9.6 (5.9)
Care stressors (0–15)	1.9 (2.4)
Social stressors (0–15)	4.9 (3.2)

Values are mean (SD). ^a^Missing in 3.7%. ^b^Comorbidities include cardiovascular, pulmonary, locomotor, neuropsychiatric, oncological and metabolic diseases. ^c^Psychiatric comorbidities include anxiety, depression and addiction. ^d^Total score consists of the sub scores of care and social stressors and two additional questions regarding COVID-19 symptoms and physical activity and relaxation.

### Association between COVID-19 stressors and depressive and anxiety symptoms

**Fig. 1:**
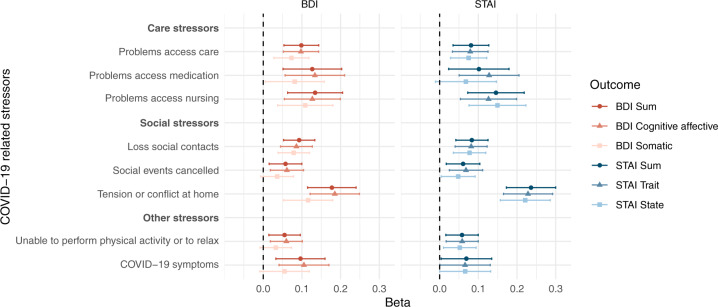
Association between COVID-19 related stressors and depressive and anxiety symptoms. Points represent the regression coefficients of the linear models and bars the 95% confidence intervals. The BDI, STAI, and their respective sub scores were standardized in order to make the estimates comparable. Models were adjusted for sex, age, disease duration, presence of comorbidities, education, living situation, region and date. *N* = 844.

A one-point increase in the COVID-19 stressors sum score was associated with a 0.04 (95% CI: 0.02–0.05) standard deviation higher BDI and a 0.03 (95% CI: 0.02–0.05) standard deviation higher STAI. The stressors sum score was also associated with higher sub scores of BDI and STAI. Care stressors (BDI beta: 0.07, 95% CI: 0.04–0.10 and STAI beta: 0.06, 95% CI: 0.04–0.09) and social stressors (BDI beta: 0.06, 95% CI: 0.04–0.08 and STAI beta: 0.06, 95% CI: 0.04–0.08) were similarly associated with both outcomes. Associations between the eight individual COVID-19 stressors and BDI and STAI are shown in Fig. 1. The highest increase in standard deviation of the outcomes was found for the stressor tension or conflict at home, followed by problems with access to nursing and problems with access to medication.

### Stratification by subgroups

**Fig. 2:**
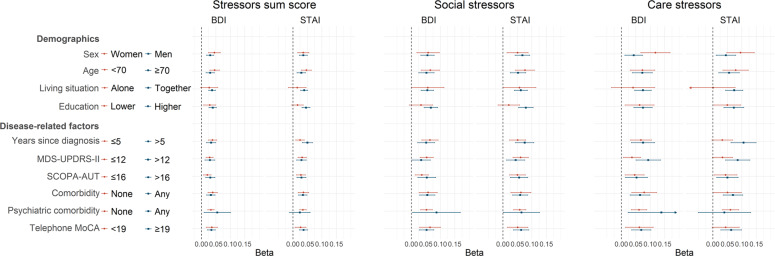
Association between sum scores of COVID-19 related stressors and depressive and anxiety symptoms stratified by demographics and disease-related factors. Points represent the regression coefficients of the linear models and bars the 95% confidence intervals. The BDI and STAI were standardized in order to make the estimates comparable. Models were adjusted for sex, age, disease duration, presence of comorbidities, education, living situation, region and date. The stratification variable was excluded for adjustments. Higher education was defined as post-secondary vocational education, pre-university education or higher. Age, disease duration, MDS-UPDRS-II, SCOPA-AUT and Telephone MoCA were dichotomized according to the median. *N* = 844.

Stratification by demographics and disease-related characteristics is shown in Fig. 2. For social stressors, larger effect estimates were found in the higher education stratum, especially for the association with STAI (beta higher education: 0.08, 95% CI: 0.05–0.10 versus beta lower education: 0.02, 95% CI: −0.02–0.06). In addition, the effect estimates of the associations between social stressors and BDI and STAI were slightly larger in people below the age of 70 years, but confidence intervals largely overlapped. For care stressors, larger effect estimates were found in women (BDI beta women: 0.12, 95% CI: 0.07–0.17 versus BDI beta men: 0.04, 95% CI: 0.01–0.07, similar for STAI), people with a longer disease duration (STAI beta ≤ 5 years: 0.03, 95% CI: 0.00–0.07 versus STAI beta > 5 years: 0.11, 95% CI: 0.06–0.15) and a higher MDS-UPDRS-II (BDI beta ≤ 12: 0.04, 95% CI: 0.01–0.07 versus BDI beta > 12: 0.09, 95% CI: 0.05–0.14, similar for STAI). Larger effect estimates were also found for care stressors in people living together and people with psychiatric comorbidities, but confidence intervals were wide and largely overlapped in these stratifications.

**Fig. 3:**
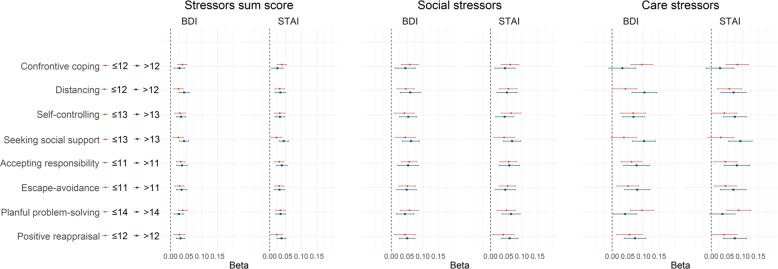
Association between sum scores of COVID-19 related stressors and depressive and anxiety symptoms stratified by coping factors. Points represent the regression coefficients of the linear models and bars the 95% confidence intervals. The BDI and STAI were standardized in order to make the estimates comparable. Models were adjusted for sex, age, disease duration, presence of comorbidities, education, living situation, region and date. The stratification variable was excluded for adjustments. All domains were dichotomized according to the median. *N* = 830, data on the coping questionnaire was missing in 1.7% because the participant could not imagine a stressful situation in the past twelve months.

Stratification by coping characteristics is shown in Fig. 3. Differences between strata were mainly found for the association between care stressors and BDI and STAI. Smaller effect estimates were found in the stratum of individuals scoring high on confrontive coping (BDI beta ≤ 12: 0.09, 95% CI: 0.06–0.13 versus BDI beta > 12: 0.03, 95% CI −0.01–0.08, similar for STAI) and planful problem solving (BDI beta ≤ 14: 0.10, 95% CI: 0.06–0.13 versus BDI beta > 14: 0.04, 95% CI 0.00–0.08, similar for STAI), whereas larger effect estimates were found in the strata of individuals scoring high on distancing (BDI beta ≤ 12: 0.04, 95% CI: 0.00–0.08 versus BDI beta > 12: 0.10, 95% CI 0.06–0.14) and seeking social support (BDI beta ≤ 13: 0.04, 95% CI: 0.00–0.08 versus BDI beta > 13: 0.10, 95% CI 0.07–0.14, similar for STAI).

### Association between COVID-19 stressors and quality of life

**Fig. 4:**
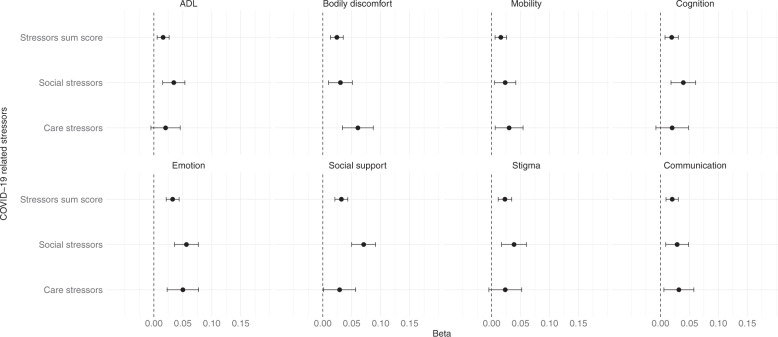
Association between sum scores of COVID-19 related stressors and quality of life. Points represent the regression coefficients of the linear models and bars the 95% confidence intervals. The PDQ-39 domains were standardized in order to make the estimates comparable. Higher PDQ-39 domain scores represent worse experienced quality of life. Models were adjusted for sex, age, disease duration, presence of comorbidities, education, living situation, region, and date. *N* = 844.

A one-point increase in the COVID-19 stressors sum score was associated with a 0.03 (95% CI: 0.02–0.04) standard deviation higher PDQ-39. Care stressors (beta: 0.05, 95% CI: 0.02–0.08) and social stressors (beta: 0.06, 95% CI: 0.04–0.08) were also associated with PDQ-39. The COVID-19 stressors sum score was associated with worse quality of life on all PDQ-39 domains (Fig. 4).

### Hypothetical interventions on COVID-19 stressors

Table 2 shows the standardized mean outcomes for seven hypothetical interventions. Intervention 1, complete removal of COVID-19 stressors in all individuals, decreased the mean BDI from 11.6 (95% CI 11.2–12.1) to 9.3 (95% CI: 8.5–10.1), STAI from 75.9 (95% CI: 74.6–77.1) to 69.6 (95% CI: 67.1–72.1), and PDQ-39 from 25.8 (95% CI 25.0–26.7) to 21.7 (95% CI: 20.1–23.2). Intervention 2, a 50% reduction of COVID-19 stressors, decreased the mean BDI to 10.5 (95% CI: 10.0–10.9), STAI to 72.7 (95% CI: 71.4–74.0), and PDQ-39 to 23.8 (95% CI: 22.9–24.6) and intervention 3, a 25% reduction of COVID-19 stressors, decreased the mean BDI to 11.1 (95% CI: 10.2–11.9), STAI to 74.3 (95% CI: 71.8–76.8), and PDQ-39 to 24.8 (95% CI: 23.3–26.4). Because social stressors were more prevalent than care stressors, intervening on social stressors resulted in the largest decrease in outcomes. In addition to the hypothetical interventions on all individuals, we modeled a 50 and 25% reduction in COVID-19 stressors in individuals with an above median stressor sum score (intervention 4 and 5, *N* = 412), and a 50 and 25% reduction in COVID-19 stressors in individuals with an above median MDS-UPDRS-II (intervention 6 and 7, *N* = 366). These hypothetical interventions decreased the BDI, STAI, and PDQ-39 to a slightly lesser extent than interventions 2 and 3. Within the targeted subgroups, the largest effect was observed for hypothetical intervention 6, which decreased the mean BDI in the subgroup with above median MDS-UPDRS-II from 14.7 (95% CI: 14.2–15.1) to 10.6 (95% CI: 9.7–11.4), STAI from 81.6 (95% CI: 80.3–82.8) to 73.1 (95% CI: 70.7–75.6) and PDQ-39 from 35.0 (95% CI: 34.2–35.9) to 24.3 (95% CI: 22.8–25.8).

**Table 2:** Population standardized means for seven hypothetical interventions on COVID-19 related stressors.

Population-wide average outcomes			
	BDI	STAI	PDQ-39
Current	11.6 (11.2–12.1)	75.9 (74.6–77.1)	25.8 (25.0–26.7)
Stressors sum score			
Intervention 1	9.3 (8.5–10.1)	69.6 (67.1–72.1)	21.7 (20.1–23.2)
Intervention 2	10.5 (10.0–10.9)	72.7 (71.4–74.0)	23.8 (22.9–24.6)
Intervention 3	11.1 (10.2–11.9)	74.3 (71.8–76.8)	24.8 (23.3–26.4)
Intervention 4	10.8 (10.3–11.2)	73.6 (72.3–74.8)	24.3 (23.5–25.2)
Intervention 5	11.2 (10.4–12.0)	74.7 (72.2–77.2)	25.0 (23.5–26.6)
Intervention 6	11.1 (10.6–11.5)	74.4 (73.1–75.7)	24.8 (23.9–25.7)
Intervention 7	11.4 (10.5–12.2)	75.1 (72.6–77.6)	25.4 (23.8–26.9)
Social stressors			
Intervention 1	9.7 (8.9–10.6)	70.3 (67.9–72.6)	22.1 (20.7–23.6)
Intervention 2	10.7 (10.2–11.1)	73.1 (71.8–74.3)	24.0 (23.1–24.8)
Intervention 3	11.2 (10.3–12.0)	74.5 (72.1–76.8)	24.9 (23.5–26.4)
Intervention 4	11.0 (10.5–11.4)	73.9 (72.6–75.2)	24.6 (23.7–25.4)
Intervention 5	11.3 (10.4–12.1)	74.8 (72.4–77.2)	25.1 (23.6–26.5)
Intervention 6	11.2 (10.7–11.6)	74.5 (73.2–75.8)	24.9 (24.0–25.7)
Intervention 7	11.4 (10.6–12.3)	75.2 (72.8–77.6)	25.4 (23.9–26.9)
Care stressors			
Intervention 1	10.7 (10.1–11.3)	73.6 (71.9–75.2)	24.6 (23.5–25.6)
Intervention 2	11.2 (10.7–11.6)	74.7 (73.4–76.0)	25.2 (24.4–26.1)
Intervention 3	11.4 (10.9–12.0)	75.3 (73.7–77.0)	25.5 (24.5–26.6)
Intervention 4	11.2 (10.8–11.7)	74.8 (73.5–76.1)	25.3 (24.4–26.1)
Intervention 5	11.4 (10.9–12.0)	75.3 (73.7–77.0)	25.5 (24.5–26.6)
Intervention 6	11.4 (11.0–11.9)	75.3 (74.0–76.6)	25.5 (24.7–26.4)
Intervention 7	11.5 (11.0–12.1)	75.6 (73.9–77.3)	25.7 (24.6–26.8)
Average outcomes for targeted subgroups			
Stressors sum score			
Current	12.8 (12.3–13.2)	78.7 (77.4–80.0)	28.2 (27.4–29.1)
Intervention 4	11.1 (10.2–11.9)	74.2 (71.7–76.7)	24.9 (23.4–26.5)
Intervention 5	11.9 (11.1–12.8)	76.5 (74.0–79.0)	26.4 (24.9–28.0)
Current	14.7 (14.2–15.1)	81.6 (80.3–82.8)	35.0 (34.2–35.9)
Intervention 6	10.6 (9.7–11.4)	73.1 (70.7–75.6)	24.3 (22.8–25.8)
Intervention 7	11.6 (10.7–12.4)	75.2 (72.7–77.7)	27.2 (25.6–28.7)

Hypothetical intervention 1 included a complete removal of COVID-19 stressors in all individuals (*N* = 844), hypothetical intervention 2 a 50% reduction of COVID-19 stressors (*N* = idem) and hypothetical intervention 3 a 25% reduction (*N* = idem). Hypothetical intervention 4 reduced COVID-19 stressors with 50% in individuals scoring above the population median (*N* = 412 for intervention on stressors sum score, *N* = 374 for intervention on social stressors, *N* = 381 for intervention on care stressors), hypothetical intervention 5 with 25% (*N* = idem). Hypothetical intervention 6 included a 50% reduction of COVID-19 stressors in people with an above median MDS-UPDRS-II (*N* = 366) and hypothetical intervention 7 a 25% reduction (*N* = idem). Models were adjusted for sex, age, disease duration, presence of comorbidities, education, living situation, region, and date. Models for intervention 6 and 7 were not adjusted for disease duration.

### Sensitivity analyses

Supplementary Fig. 2 shows the association between the eight COVID-19 stressors and BDI and STAI, stratified by questionnaire completion date. Most associations were apparent across the three time strata. Supplementary Fig. 3 shows the main results after excluding participants with psychiatric comorbidities, which are similar to the results shown in Fig. 1. In an additional analysis of the Personalized Parkinson Project (PPP) data, we found an association between the COVID-19 stressor sum score (0–25) and pre-COVID-19 BDI (beta: 0.06, 95% CI: 0.03–0.08), STAI (beta: 0.05, 95% CI: 0.02–0.07) and PDQ-39 (beta: 0.06, 95% CI: 0.03–0.09). Supplementary Table 1 shows the results of the subsequent analyses of the association between the COVID-19 stressor sum score and PSS, PAS and RRS, adjusted for pre-COVID-19 BDI, STAI and PDQ-39. The stressor sum score was still associated with PSS (beta: 0.04, 95% CI: 0.02–0.06), PAS (beta: 0.04, 95% CI: 0.02–0.07) and RSS (beta: 0.02, 95% CI: 0.00–0.04) after adjustment for pre-COVID-19 scores. Our final sensitivity analysis included a comparison of COVID-19 stress and the relation between this stress and depressive and anxiety symptoms and quality of life between participants with and without parkinsonism in the Rotterdam Study. We found that COVID-19 stress was somewhat lower in people with parkinsonism (beta: −0.61, 95% CI: −1.59–0.38). However, as can be seen in Supplementary Fig. 4, the association between COVID-19 stress and depressive and anxiety symptoms and quality of life seemed slightly larger in people with parkinsonism. Nevertheless, because of the limited number of people with parkinsonism in these analyses, the confidence intervals were very wide and the differences between the groups thus uncertain.

## Discussion

In this cross-sectional study among people with PD, we found that the association between COVID-19 stressors and mental health was more pronounced in women, highly educated people, people with advanced PD and people prone to distancing or seeking social support. Effects were less pronounced in people prone to confrontive coping or planful problem solving. Our results suggest that intervening on COVID-19 stressors in people with more advanced PD might result in clinically important improvements of mental health and quality of life.

Our findings must be interpreted in light of the limitations of this study. First, the cross-sectional nature of this study impedes the interpretation of the direction of effect. Reverse causation could importantly influence the interpretation of our results as people with pre-existing depressive or anxiety symptoms might report a larger influence of COVID-19 stressors on mental health. Excluding people with psychiatric comorbidities did not meaningfully alter our results, although pre-existing depression or anxiety was self-reported and thus might have been underdiagnosed. Moreover, in a post-hoc analysis with the PPP data, we still observed an effect of COVID-19 stressors on mental health after adjustment for pre-COVID-19 depressive and anxiety symptoms and quality of life. Second, we did not study whether the associations between COVID-19 stressors and mental health differed in persons with PD as compared to a reference population. As a sensitivity analysis, we compared the effects between people with and without parkinsonism in the Rotterdam Study. Of note, the questionnaires used in that cohort differed somewhat from those used in the PRIME-NL study and the sample of people with parkinsonism in the Rotterdam Study was small. Third, the shown associations were similar for state and trait anxiety, whereas a more evident effect of COVID-19 stressors is to be expected on state anxiety. This might suggest that the construct validity of the STAI for differentiating state and trait anxiety is low^15^. The approach by Yule et al^16^. to ask participants to complete the state anxiety questions keeping in mind the situation since the COVID-19 pandemic and the trait anxiety questions keeping in mind the situation before the COVID-19 pandemic might help to overcome this issue. Finally, although we extensively adjusted for potential confounders, residual confounding might still affect the results.

A strength of this study includes the remote data collection, which made it possible to measure multiple clinically relevant domains of wellbeing in a large sample of people with PD during the COVID-19 pandemic. The sample represents the overall population of people with PD treated in community hospitals and not solely included patients treated in specialized centers. Furthermore, we performed thorough analyses, not only showing the overall association between COVID-19 stressors and mental health, but also investigating associations in subgroups and potential effects of realistic hypothetical interventions. Finally, we performed several sensitivity analyses to determine the probability that reverse causation drives our results. Inconsistent results have been described regarding the effect of the COVID-19 pandemic on mental health of people with PD^6,17–19^. Several studies have compared depressive or anxiety symptoms between people with PD and controls^4,5,20–23^ and concluded that mental health during the pandemic is worse in people with PD^4,5,21–23^. However, this effect is not specific to the COVID-19 pandemic, as a higher prevalence of depression and anxiety is observed in people with PD regardless of the COVID-19 pandemic^1,2^. In most previous studies, at least part of the population of people with PD experienced worsening of motor or non-motor symptoms^5,12,20,23–32^. Our study aimed to specifically determine which subgroups of people with PD might be most vulnerable to the effect of the COVID-19 pandemic on mental health.

Previous studies found women and younger individuals to be more vulnerable to depressive and anxiety symptoms during the COVID-19 pandemic^22,32,33^ and our results point into a similar direction. Surprisingly, we found that the association between social stressors and mental health was more evident in highly educated people, specifically for the association between the stressor cancellation of social events with anxiety. Similarly, a study among 1,143 U.S. adults found that depressive symptoms increased more during the COVID-19 pandemic in higher than lower educated people^34^. This effect could not be explained by COVID-related knowledge or job loss, but was suggested to result from expectations about available resource^34^, an explanation that warrants further investigation. Our disease-related stratifications showed that the association between care stressors and anxiety was most pronounced in people with more advanced PD, which is similar to previous observations^23,33^. This effect is not surprising given that people with advanced PD need more care and might thus also experience more anxiety symptoms upon cancellation of care. Interestingly, our results suggested that the effect of access to medication on anxiety seemed to become greater over time. However, this observation needs to be replicated in future studies.

Some coping strategies, such as approach coping, positive reframing, acceptance and humor, have been associated with better mental health during the COVID-19 pandemic^35,36^, whereas avoidant coping^36,37^, self-blame, venting, behavioral disengagement, and self-distraction^35^ have been associated with worse mental health. We investigated specifically the association between COVID-19 stressors and mental health among people with different coping strategies. Our results support previous findings that actively trying to change the situation could be a protective coping strategy, while avoidant coping could be detrimental. Yet, contrary to previous studies, we found smaller effect sizes in people scoring high on confrontive coping and larger effect sizes in people prone to seeking social support. These findings may represent PD or pandemic-specific effects, but this needs to be investigated further.

Hypothetical interventions are important from a public health point of view because they take into account both the prevalence and effect size of a risk factor. Our simulated hypothetical intervention which removed all COVID-19 stressors resulted in improved depressive and anxiety symptoms and quality of life on a population level. However, we think complete removal of COVID-19 stressors in all people with PD is not feasible in practice because of financial and time constraints and because interventions to reduce stress will not be 100% effective. Thus, we also simulated the effect of a 50 and 25% reduction in COVID-19 stressors in the entire study population and in targeted groups. We found that a hypothetical 50% reduction in COVID-19 stressors in people with an above median MDS-UPDRS-II (*N* = 366) decreased BDI with about four points, STAI with about eight points and PDQ-39 with about ten points in the targeted groups. In the entire study population, this targeted intervention showed much smaller effects, only a decrease of half to one and a half point. The literature is inconclusive about the minimum clinically important difference (MCID) for our outcomes, but previously reported MCIDs ranged from 3 to 9 for the BDI^38,39^, was 10 for the STAI^40^ and 5 for the PDQ-39^41^. These MCIDs suggest that a 50% reduction, or even only a 25% reduction in COVID-19 stressors in people with more advanced PD could potentially result in clinically meaningful improvements in mental health or quality of life in this group, but not on a population level. Since we only considered eight COVID-19 stressors and thus might have missed important stressors such as economic loss and sleep, the effects of hypothetical interventions could increase if a more elaborate set of COVID-19 stressors would be considered.

Although real-life interventions might only reduce part of the COVID-19 stress, because not all stress is modifiable, there are several interventions for which a substantial reduction in stress might be expected. A potential intervention to decrease care stressors during the pandemic is the provision of telemedicine, for instance by virtual consultations^42,43^. In our study population, virtual consultations with healthcare providers were infrequent and much lower than described in a previous study^13^. Only 3% of participants had video contact with their neurologist, 1% with the nurse, 7% with the physiotherapist and 5% with the speech therapist. Enhancing the use of video consultations could importantly improve access to care and reduce the potential impact of care stressors on mental health and quality of life^6^. Social stressors were more prevalent in our study and thus our hypothetical interventions on these stressors showed highest potential of effect. Social prescribing and virtual social support groups have been suggested as a way to keep connected, reduce social isolation and loneliness and inform people with PD on topics such as stress management and resilience^44,45^. Furthermore, online classes, such as online dance classes, provide not only the possibility to stay physically active, but also to connect with others^46,47^. Participants in online dance classes reported reduced anxiety and stress and improved mood as a result of these classes^47^. However, to target the most important social stressor influencing depressive and anxiety symptoms in our study, tension or conflict at home, a more personalized approach will be needed. Potentially, social workers could play an important role in reducing social stressors, since supportive counseling of both patients and their relatives by social workers is targeted at maintaining psychological wellbeing, preventing social isolation and preserving relationships with friends and family^48,49^. Tailored interventions will be necessary to reduce the effect both of care and social stressors on mental health and quality of life in people with PD.

Further research is needed to better understand the possible effects of tailored interventions on stressors to improve mental health. First, longitudinal studies are necessary to confirm our findings and further rule out reverse causation. Second, the feasibility of real-life interventions to reduce COVID-19 stressors with 25% or 50% must be evaluated. Third, differential effects on subgroups of people with PD not sufficiently represented by this study, for instance people in assisted living, must be considered. Finally, we showed that coping strategies influence the association of stressors with mental health, but the ability of people with PD to cope with stress might also affect the outcomes regardless of the stress level. The independent effect of coping on mental health and quality of life warrants further investigation.

Insights from this cross-sectional study help to inform tailored care interventions to subgroups of people with PD most vulnerable to the impact of COVID-19. Intervening on COVID-19 stressors in people with advanced PD might result in clinically important improvements in mental health and quality of life.

## Methods

### Study Population

This cross-sectional study was embedded within the Proactive and Integrated Management and Empowerment in Parkinson’s Disease – Netherlands (PRIME-NL) study, a prospective cohort study of persons with parkinsonism and their caregivers^50^. The PRIME-NL study is conducted in two regions: the PRIME Parkinson care region, including four community hospitals that collaborate directly with the Radboud University Medical Center, and the usual care region, including 60 community hospitals outside of the PRIME region. The baseline measurement of the PRIME-NL study encompassed 988 participants included from February to December 2020. The current study focused on 844 participants with PD (95.1% of parkinsonisms in this study) who completed a questionnaire on COVID-19 stressors. Participants in the current study completed the baseline PRIME-NL questionnaire between April 14^th^ 2020 and February 25^th^ 2021.

Participants represented the broad spectrum of people with PD who are treated in community hospitals. Eligible participants visited the outpatient clinic at least once a year and were not treated in tertiary hospitals. Patients were recruited through the ParkinsonNEXT database^51^, the Dutch parkinsonism patient association^52^ and through neurologists in the PRIME Parkinson care region. PD diagnosis was self-reported and confirmed by a letter of the general practitioner or neurologist.

The PRIME-NL study has been approved by the Ethical Board of the Radboud University Medical Center. All participants provided digital or written informed consent before inclusion in the PRIME-NL study.

### Questionnaire-based data

Participants self-administered questionnaires electronically or, if unable to do so, were provided with either a paper-based self-administration or a telephone-based administration.

Since April 2020, the PRIME-NL questionnaire included eight statements about different situations that could have occurred during the COVID-19 pandemic, based on the DynaCORE questionnaire^53^. The question that accompanied each statement was: ‘Could you indicate how you experience or experienced these situations because of the COVID-19 pandemic?’ Each question was scored on a six-point Likert-scale ranging from ‘this situation did not occur’ to ‘very troublesome’. A social stressors score was calculated, summarizing statements about loss of social contacts, cancellation of social events and tension or conflict at home, and a care stressors score, summarizing statements about problems with access to care, medication and nursing. Two additional COVID-19 stressors, regarding possible COVID-19 symptoms and physical activity and relaxation, were not included in the sub scores, but were summed up in the stressors sum score including all eight items. A detailed description of the questionnaire can be found in Supplementary Table 2. At the moment the questionnaire was sent out, testing for COVID-19 was not yet widely available in the Netherlands, we thus do not have information on COVID-19 diagnoses.

Depressive symptoms were measured using the Beck Depression Inventory (BDI)^54^. Sum scores were calculated as well as the affective-cognitive (item 1 to 14) and somatic sub score (item 15 to 21). Anxiety symptoms were measured using the State-Trait Anxiety Inventory (STAI)^55^. Sum scores and State (event-related) and Trait (personality-related) scores were calculated. The BDI and STAI were strongly correlated (Pearson’s R 0.68) and measured two partially overlapping entities of the broader concept of mental health. In order not to make any prior assumptions about agreements in effects on both outcomes, we analyzed the BDI, STAI and their sub scores separately. Quality of life was measured using the Parkinson’s Disease Quality of Life Questionnaire (PDQ-39)^56^, which was summarized into the domains mobility, activities of daily living, emotional well-being, stigma, social support, cognition, communication, and bodily discomfort. The PDQ-39 sum score was calculated as the mean of each domain. Higher scores on the BDI, STAI, and PDQ-39 represent worse depressive and anxiety symptoms and poorer quality of life.

Comorbidities were self-reported and included cardiovascular, pulmonary, locomotor, neuropsychiatric, oncological and metabolic diseases. Motor aspects of daily living were assessed using the Movement Disorders Society Unified Parkinson Disease Rating Scale Part II (MDS-UPDRS-II)^57^. Non-motor symptoms were measured with the Scales for Outcomes in Parkinson’s Disease – autonomic dysfunction (SCOPA-AUT)^58^, excluding the sexual domain. To be able to compare the SCOPA-AUT with other studies, we divided the total score by 63 (current maximum score) and multiplied it by 69 (original maximum score). Cognition was assessed using a shortened version of the Montreal Cognitive Assessment (Telephone MoCA)^59^, excluding questions about location. For comparability, we added two points to the total score for all individuals. Coping strategies were determined with the Ways of Coping questionnaire (WCQ)^60^. Eight coping domains were created from this questionnaire: confrontive coping, distancing, self-controlling, seeking social support, accepting responsibility, escape-avoidance, planful problem-solving and positive reappraisal (Supplementary Table 3)^61^.

Linear regression models were fitted with individual COVID-19 stressors and stressors sum scores as determinants and standardized BDI, STAI and PDQ-39 as outcomes. COVID-19 stressors were included as continuous variables and models were adjusted for sex, age, disease duration, presence of comorbidities, education, living situation, region and date.

Stratifications were performed on three levels: demographics, disease-related and coping characteristics. Demographics included sex, age, living situation and education. Disease-related characteristics included disease duration, motor symptoms, non-motor symptoms, comorbidities, psychiatric comorbidities and cognition. Coping characteristics included the eight domains of the WCQ. Continuous variables were dichotomized for stratifications according to the median. All stratified analyses were adjusted as described above, excluding the stratification variable as covariate.

Simulating hypothetical interventions provides the possibility to test the potential effect of interventions by taking into account both the prevalence and effect size of COVID-19 stressors. We calculated standardized means of BDI, STAI and PDQ-39 for seven hypothetical interventions, including complete removal and a 50% or 25% reduction of COVID-19 stressors in all individuals (intervention 1, 2 and 3) and a 50% or 25% reduction of COVID-19 stressors in individuals scoring above the population median (intervention 4 and 5). Intervention 6 and 7 were based on the stratification results and included a 50% or 25% reduction of COVID-19 stressors in people with an above median MDS-UPDRS-II. Standardized means were obtained using the parametric G-formula method^62^ and included expanding the dataset, outcome modeling, prediction and standardization by averaging. Similar methods have been used by several previous studies and the validity of the estimates is dependent on the same assumptions as standard methods^62–67^. Models were adjusted as described above, except for the models of interventions 6 and 7 which were not adjusted for disease duration. Outcomes were studied on their original scale.

**Fig. 5:**
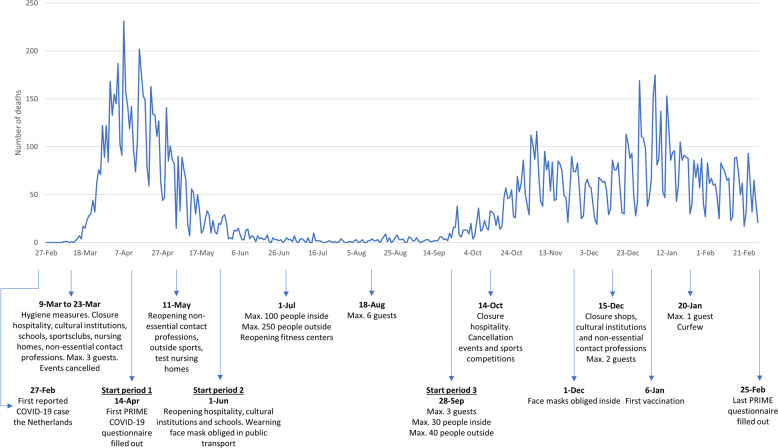
Timeline of COVID-19 related deaths and restrictions in the Netherlands. Data derived from: https://data.rivm.nl/covid-19/ and https://www.rijksoverheid.nl/actueel/nieuws.

Four sensitivity analyses were performed. In a first sensitivity analysis, we repeated the analysis of the eight individual COVID-19 stressors for three time strata of the baseline assessment with differing intensity of the COVID-19 restrictions issued by the government: from April 14th to June 1^st^ (period 1), from June 1^st^ to September 28th (period 2) and from September 28^th^ to February 25^th^ (period 3). A timeline of the COVID-19 restrictions in the Netherlands can be found in Fig. 5. Because we recognize the possibility of reverse causation as an important limitation of our study, the following two sensitivity analyses were performed to mitigate this concern. In the second sensitivity analysis, we repeated the analysis of the eight COVID-19 stressors in a sample excluding participants with self-reported psychiatric comorbidities. In a third sensitivity analysis, we performed a post-hoc analysis using data of a complementary cohort (Personalized Parkinson Project, PPP), which has performed measurements of mental health during and shortly before the COVID-19 pandemic. In this cohort, we determined whether the COVID-19 stressor sum score was associated with pre-COVID-19 depressive and anxiety symptoms and quality of life and whether the relationship between the COVID-19 stressor sum score and the outcomes persisted after adjustment for pre-COVID-19 mental health. In a final sensitivity analysis, using data of the population-based Rotterdam Study, we studied whether COVID-19 stress was higher in people with parkinsonism and whether the relation between COVID-19 stress and depressive and anxiety symptoms and quality of life was stronger in participants with than without parkinsonism. A more detailed description of the PPP, the Rotterdam Study, and used methods can be found in Supplementary Note 1.

In this paper, we will not dichotomize the effects into ‘significant’ and ‘non-significant’. Instead, we will show effect estimates and confidence intervals in order to describe a range of effect estimates that are compatible with the data^68^. All analyses were performed using R version 3.6.2.

### Reporting summary

Further information on research design is available in the Nature Research Reporting Summary linked to this article.

## Supplementary information

Supplementary information

Reporting Summary

## Data Availability

Applications for PRIME-NL data should be directed towards the corresponding author.

## Code availability

The code is available in the Supplementary Information.
