# Supplementary material for: Mental health in people with Parkinson’s disease during the COVID-19 pandemic: potential for targeted interventions?
Source: NPJ Parkinsons Dis. 2021 Oct 28;7:95. doi: 10.1038/s41531-021-00238-y (PMC8553848; doi:10.1038/s41531-021-00238-y)
Supplement: Supplementary file 1 — Supplementary information [file 41531_2021_238_MOESM1_ESM.pdf]

nature portfolio

Corresponding author(s): S.K.L. Darweesh

Last updated by author(s): 09-07-2021

Reporting Summary

Nature Portfolio wishes to improve the reproducibility of the work that we publish. This form provides structure for consistency and transparency in reporting. For further information on Nature Portfolio policies, see our Editorial Policies and the Editorial Policy Checklist.

Statistics

For all statistical analyses, confirm that the following items are present in the figure legend, table legend, main text, or Methods section.

n/a	Confirmed	
☐	☒	The exact sample size (*n*) for each experimental group/condition, given as a discrete number and unit of measurement
☐	☒	A statement on whether measurements were taken from distinct samples or whether the same sample was measured repeatedly
☒	☐	The statistical test(s) used AND whether they are one- or two-sided<br>*Only common tests should be described solely by name; describe more complex techniques in the Methods section.*
☐	☒	A description of all covariates tested
☐	☒	A description of any assumptions or corrections, such as tests of normality and adjustment for multiple comparisons
☐	☒	A full description of the statistical parameters including central tendency (e.g. means) or other basic estimates (e.g. regression coefficient) AND variation (e.g. standard deviation) or associated estimates of uncertainty (e.g. confidence intervals)
☒	☐	For null hypothesis testing, the test statistic (e.g. *F*, *t*, *r*) with confidence intervals, effect sizes, degrees of freedom and *P* value noted<br>*Give P values as exact values whenever suitable.*
☒	☐	For Bayesian analysis, information on the choice of priors and Markov chain Monte Carlo settings
☐	☒	For hierarchical and complex designs, identification of the appropriate level for tests and full reporting of outcomes
☐	☒	Estimates of effect sizes (e.g. Cohen's *d*, Pearson's *r*), indicating how they were calculated

Our web collection on statistics for biologists contains articles on many of the points above.

Software and code

Policy information about availability of computer code

Data collection	Data of the PRIME-NL, Personalized Parkinson Project and Rotterdam Study were used.
Data analysis	R version 3.6.2 was used for all analyses

For manuscripts utilizing custom algorithms or software that are central to the research but not yet described in published literature, software must be made available to editors and reviewers. We strongly encourage code deposition in a community repository (e.g. GitHub). See the Nature Portfolio guidelines for submitting code & software for further information.

Data

Policy information about availability of data

All manuscripts must include a data availability statement. This statement should provide the following information, where applicable:

- Accession codes, unique identifiers, or web links for publicly available datasets
- A description of any restrictions on data availability
- For clinical datasets or third party data, please ensure that the statement adheres to our policy

Applications for PRIME-NL data should be directed towards the corresponding author.

Field-specific reporting

Please select the one below that is the best fit for your research. If you are not sure, read the appropriate sections before making your selection.

☒ Life sciences ☐ Behavioural & social sciences ☐ Ecological, evolutionary & environmental sciences

For a reference copy of the document with all sections, see nature.com/documents/nr-reporting-summary-flat.pdf

Life sciences study design

All studies must disclose on these points even when the disclosure is negative.

Sample size	The PRIME-NL study aimed to include a total of 1200 participants with parkinsonism. At baseline, 988 participants were actually included, of which 844 were used for the analyses in the current sample. The sample size was not specifically determined for the current study.
Data exclusions	The sample included only participants with Parkinson's disease (95.1%) because of the very small sample if participants with other causes of parkinsonism. Furthermore, participants who completed the questionnaire before April 14, 2020 were excluded because they did not fill out the COVID-19 questionnaire yet.
Replication	Data of the Personalized Parkinson Project was used to replicate our findings and assess the possibility of reverse causation.
Randomization	No randomization was performed, we adjusted models for the following covariates: sex, age, disease duration, presence of comorbidities, education, living situation, region and date.
Blinding	The described study was an observational study which did not include blinding of participants or researchers.

Reporting for specific materials, systems and methods

We require information from authors about some types of materials, experimental systems and methods used in many studies. Here, indicate whether each material, system or method listed is relevant to your study. If you are not sure if a list item applies to your research, read the appropriate section before selecting a response.

Materials & experimental systems

n/a	Involved in the study	
☒	☐	Antibodies
☒	☐	Eukaryotic cell lines
☒	☐	Palaeontology and archaeology
☒	☐	Animals and other organisms
☐	☒	Human research participants
☒	☐	Clinical data
☒	☐	Dual use research of concern

Methods

n/a	Involved in the study	
☒	☐	ChIP-seq
☒	☐	Flow cytometry
☒	☐	MRI-based neuroimaging

Human research participants

Policy information about studies involving human research participants

Population characteristics	The mean (SD) age of the participants was 70.3 (7.8) years and 321 (38.0%) participants were women. Most participants lived together with a partner or child (84.4%) and 9.1% had paid employment. Participants were diagnosed with PD at a mean (SD) age of 64.0 (9.1) years.
Recruitment	Patients were recruited through the ParkinsonNEXT database, the Dutch parkinsonism patient association and through neurologists in the PRIME Parkinson care region. Eligible participants visited the outpatient clinic at least once a year and were not treated in tertiary hospitals.
Ethics oversight	The PRIME-NL study has been approved by the Ethical Board of the Radboud University Medical Center.

Note that full information on the approval of the study protocol must also be provided in the manuscript.
