# Supplementary material for: Mental health in people with Parkinson’s disease during the COVID-19 pandemic: potential for targeted interventions?
Source: NPJ Parkinsons Dis. 2021 Oct 28;7:95. doi: 10.1038/s41531-021-00238-y (PMC8553848; doi:10.1038/s41531-021-00238-y)
Supplement: Supplementary file 2 — Reporting Summary [file 41531_2021_238_MOESM2_ESM.pdf]

Supplementary Figure 1. Average cross-sectional BDI, STAI and PDQ-39 scores by completion date questionnaire

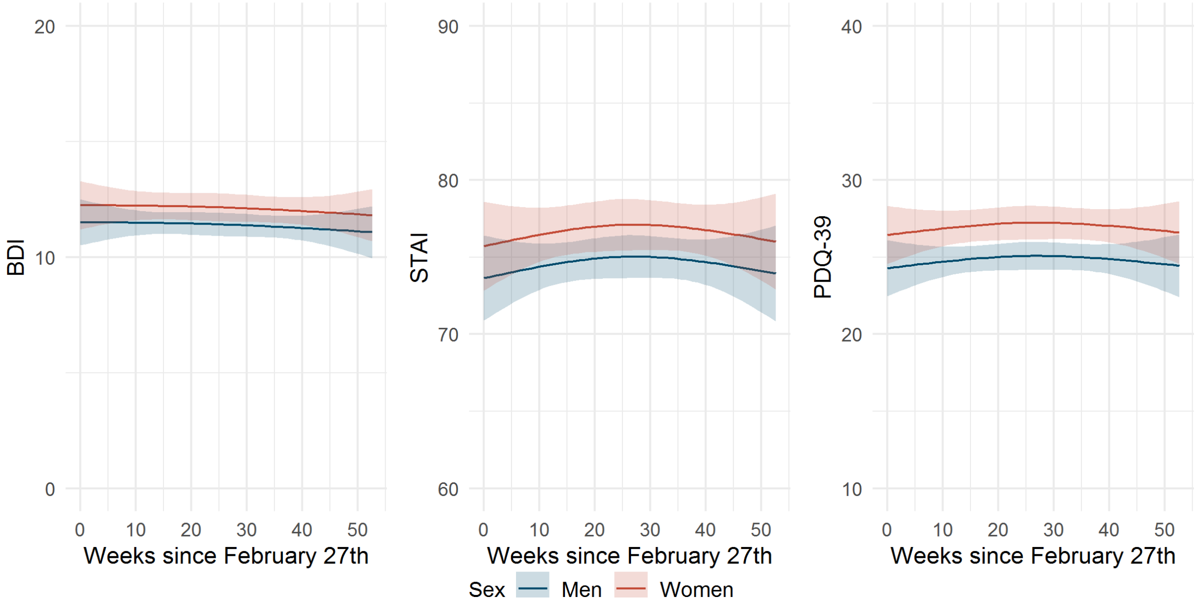

Mean outcomes since February 27th, when first COVID-19 case was reported in the Netherlands. All participants of the PRIME-NL study with Parkinson's disease (N = 940) were included in the models for this figure. Models were adjusted for sex, age, disease duration, presence of comorbidities, education, living situation, region and date.

Supplementary Figure 2. Sensitivity analysis stratified by the period of completion baseline questionnaire

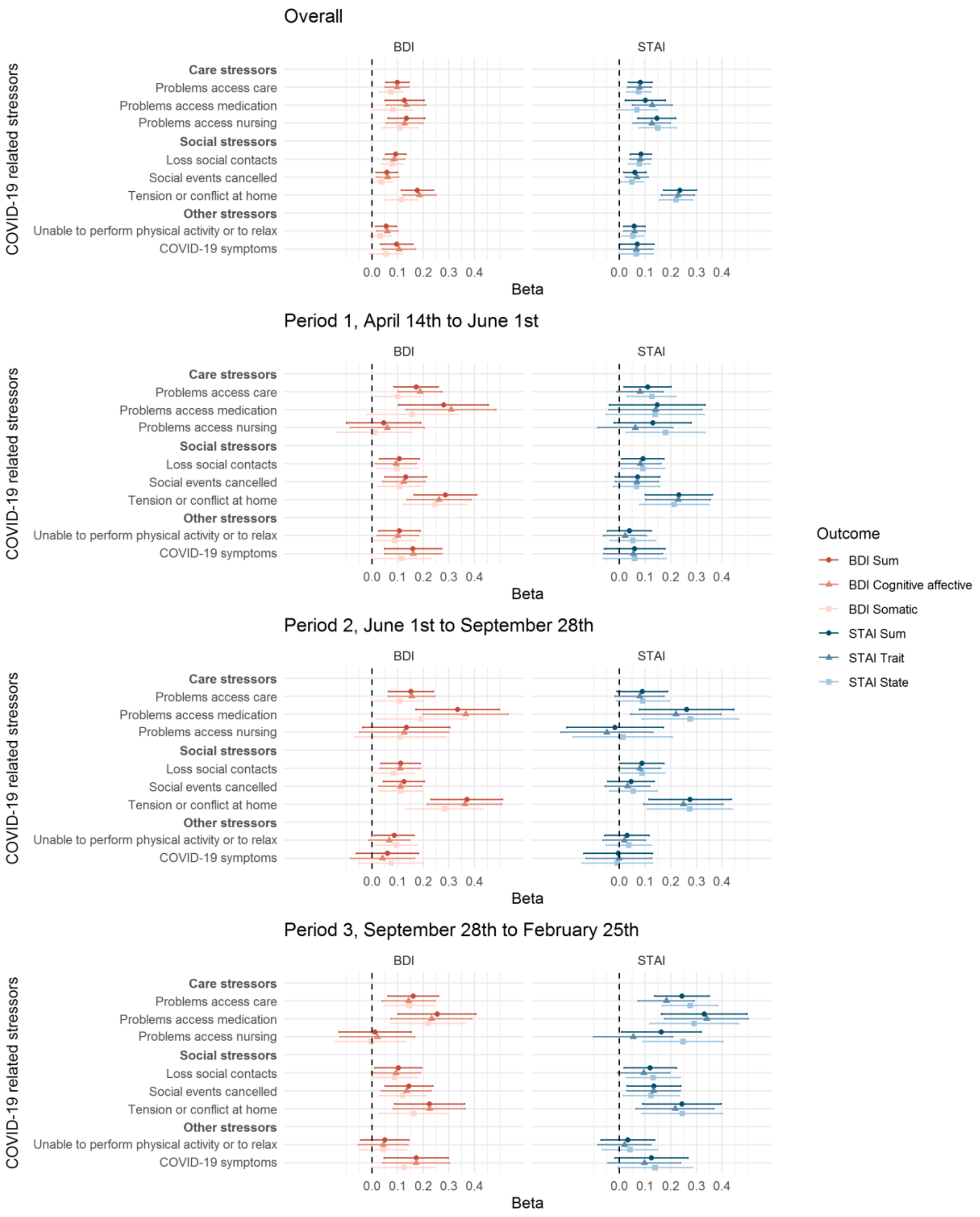

Points represent the regression coefficients of the linear models and bars the 95% confidence intervals. The BDI and STAI were standardized in order to make the estimates comparable. Models were adjusted for sex, age, disease duration, presence of comorbidities, education, living situation, region and date. N = 844.

Supplementary Figure 3. Sensitivity analysis excluding participants with psychiatric comorbidities

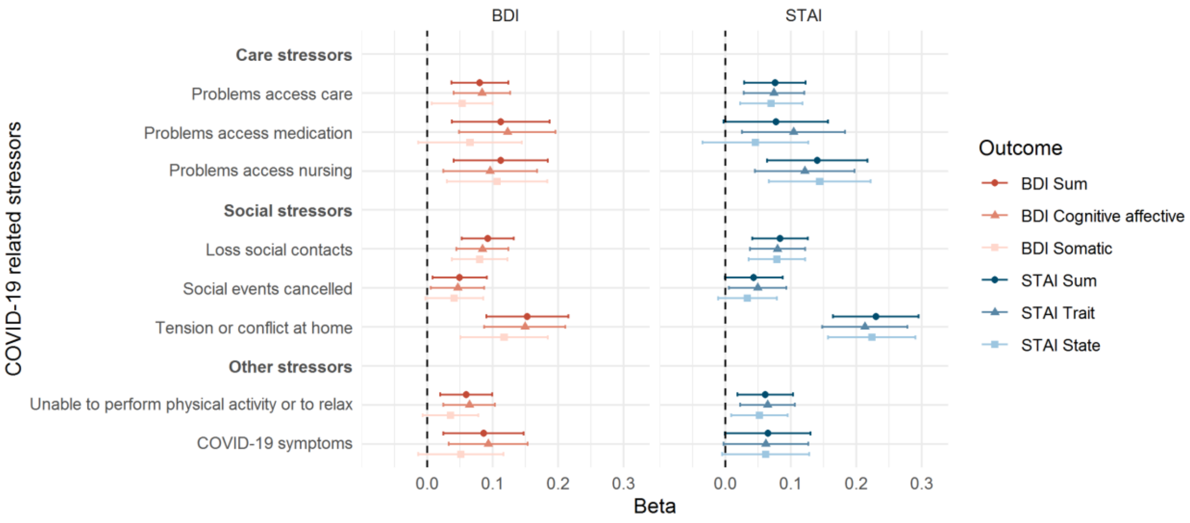

Psychiatric comorbidities were defined as anxiety, depression or addiction. Points represent the regression coefficients of the linear models and bars the 95% confidence intervals. The BDI and STAI were standardized in order to make the estimates comparable. Models were adjusted for sex, age, disease duration, presence of comorbidities, education, living situation, region and date. N = 774.

Supplementary Table 1. Association between COVID-19 stressor sum score and PSS, PAS and RRS in the Personalized Parkinson Project.

	**Model 1**	**Model 2**	**Model 3**
PSS	0.08 (0.06-0.11)	0.05 (0.03-0.07)	0.04 (0.02-0.06)
PAS	0.08 (0.06-0.11)	0.05 (0.03-0.07)	0.04 (0.02-0.07)
RSS	0.06 (0.03-0.08)	0.02 (0.00-0.04)	0.02 (0.00-0.04)

Outcomes were standardized in order to make the estimates comparable. Model 1 was adjusted for sex, age, disease duration, presence of comorbidities, education, living situation and date (n = 336). Model 2 was additionally adjusted for BDI and STAI (n = 333). Model 3 was adjusted as model 2 with an additional adjustment for PDQ-39 (n = 325).

Supplementary Figure 4. Association between COVID-19 stress and depressive and anxiety symptoms and quality of life stratified for parkinsonism in the Rotterdam Study

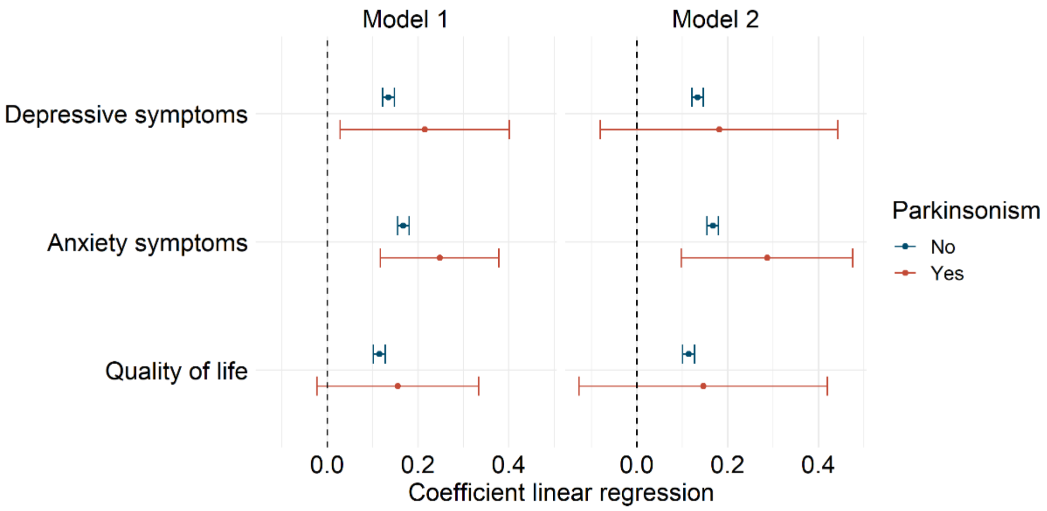

Outcomes were standardized in order to make the estimates comparable, higher scores represent worse outcomes. Model 1 was adjusted for sex and age. Model 2 was additionally adjusted for presence of comorbidities, education, living situation and date. Effect estimates model 2: depressive symptoms (beta no parkinsonism: 0.13, 95% CI: 0.12-0.15, beta parkinsonism: 0.18, 95 CI: -0.08-0.44), anxiety symptoms (beta no parkinsonism: 0.17, 95% CI: 0.15-0.18, beta parkinsonism: 0.29, 95 CI: 0.10-0.48), quality of life (beta no parkinsonism: 0.11, 95% CI: 0.10-0.13, beta parkinsonism: 0.15, 95 CI: -0.13-0.42).

Supplementary Table 2. Overview of COVID-19 stressors

Categorisation of stressors		Stressors	Translated statements questionnaire
COVID-19 stressor sum score	Care stressors	Problems access care	Problems with access to care
		Problems access mediation	Problems with access to medication
		Problems access nursing	Problems with access to nursing
	Social stressors	Loss social contacts	Loss of social contacts
		Social events cancelled	Social events which are cancelled
		Tension or conflict at home	Tension or conflict at home
		Unable to perform physical activity or to relax	Not being able to perform physical activity or to relax
		COVID-19 symptoms	Showing COVID-19 symptoms or symptoms that could be related to COVID-19

Question that accompanied the statements: ‘Could you indicate how you experience or experienced these situations because of the COVID-19 pandemic?’

Supplementary Table 3. Definition of coping strategies

Coping strategy	Definition
Confrontive coping	Aggressive efforts to alter the situation. Suggests a degree of hostility and risk-taking
Distancing	Efforts to detach oneself and creating a positive outlook
Self-controlling	Efforts to regulate one's own feelings and actions
Seeking social support	Efforts to seek informational support, tangible support and emotional support
Accepting responsibility	Acknowledges one's own role in the problem with a concomitant theme of trying to put things right
Escape-avoidance	Wishful thinking and behavioural efforts to escape or avoid
Planful problem-solving	Deliberate problem-focused efforts to alter the situation coupled with an analytic approach to solving the problem
Positive reappraisal	Efforts to create positive meaning by focusing on personal growth, it also has a religious tone

Definitions adopted from Folkman et al.[62]

Supplementary Note 1. Methods Personalized Parkinson Project and Rotterdam Study

Personalized Parkinson Project

The Personalized Parkinson Project (PPP) is a longitudinal observational study including three annual visits at the Radboud University Medical Center in Nijmegen, the Netherlands.[64] At inclusion, participants of the PPP had a disease duration of 5 years or less. All participants of the PPP (~ 500) were sent an invitation for an additional online COVID-19 questionnaire. Informed consent for participation in this add-on COVID-19 study was obtained electronically. More details of the COVID-19 questionnaire have been described previously.[12]

The PPP COVID-19 study included eighteen questions about COVID-19 related stressors. Each question was scored on a six-point Likert-scale ranging from 'this situation did not occur' to 'very troublesome'. Five stressors were similar to the COVID-19 stressors in the PRIME-NL study and included: having COVID-19 symptoms or symptoms that could be related; problems with access to healthcare, medication or sanitation; loss of social contact and social events; not able to perform physical activity or leisure activities as usual; and tensions at home or family conflict. The scores of these five stressors were summarized into a stressors sum score (0-25). The COVID-19 questionnaire also included the Perceived Stress Scale (PSS), the Parkinson Anxiety Scale (PAS) and the Ruminative Response Scale (RRS), which were used as measures for mental health in the current study. Covariate information was obtained from the most recent regular PPP visit (on average half a year before the COVID-19 questionnaire), which also included the BDI, STAI and PDQ-39.

We standardized the PSS, PAS, RRS, BDI, STAI and PDQ-39 to make the outcomes comparable, higher scores represent worse outcomes. To assess the potential for reverse causation, we first modelled the relation between the COVID-19 stressors sum score and the BDI, STAI and PDQ-39 during the latest pre-COVID-19 PPP visit. This linear regression model was adjusted for sex, age, disease duration, presence of comorbidities, education, living situation and date. Subsequently, we modelled the relation between the COVID-19 stressors sum score and the PSS, PAS and RRS obtained during the COVID-19 pandemic, adjusting the model for the covariates described above (model 1). Finally, we adjusted the model additionally for pre-COVID-19 BDI and STAI (model 2) and pre-COVID-19 BDI, STAI and PDQ-39 (model 3).

We included 336 PPP participants in the current analyses, 333 of whom had also data available on BDI and STAI and 325 on PDQ-39. The mean age of the 336 included participants at the date of filling out the COVID-19 questionnaire was 62.8 (9.1) years and 39% were women. The average age at diagnosis was 59.0 (9.1) years.

Rotterdam Study

The Rotterdam Study is a longitudinal population-based cohort study in the Rotterdam district Ommoord.[65] In April 2020, a paper-based COVID-19 questionnaire was sent out to the Rotterdam Study participants. This questionnaire included the following question about COVID-19 stress: 'How much did you worry about the COVID-19 pandemic in the last 14 days?', which was measured on a scale from 1 to 10. Furthermore, the questionnaire included the CES-D-10 to measure depressive symptoms, the HADS-A to measure anxiety symptoms and a question about self-rated quality of life on a scale from 1 to 10. Covariate information was obtained from this questionnaire as well.

The case finding approach for parkinsonism in the Rotterdam Study has been previously described in detail.[66] In short, participants with parkinsonism at baseline were identified by self-reporting, assessment of antiparkinsonian drug use and evaluation of signs of parkinsonism at neurologic screening. Subsequently, participants identified with possible parkinsonism at baseline were clinically examined by a physician with experience in neurological disorders. During follow-up, continuous monitoring of clinical records and medication use, self-reporting and in-person screening for parkinsonism were used to identify incident cases. Medical records of screen-positives were evaluated by a panel led by an experienced neurologist. Data on parkinsonism status was available until 01-01-2018, events after this date were not considered. Because of the low number of participants with PD who filled out the COVID-19 questionnaire, we studied parkinsonism instead of PD.

We standardized the CES-D-10, HADS-A and quality of life to make the outcomes comparable, higher scores on the outcomes represent worse depressive symptoms, anxiety symptoms and quality of life, respectively. We first determined whether self-reported COVID-19 stress differed between participants with and without parkinsonism using linear regression models. Subsequently, we modelled the relation between COVID-19 stress and the outcomes CES-D-10, HADS-A and quality of life in people with and without parkinsonism separately using linear regression models. Because of the low number of participants with parkinsonism, model 1 was adjusted only for age and sex and model 2 additionally for presence of comorbidities, education, living situation and date.

The sample included a total of 4,059 participants with a mean age of 74.7 (8.6) years and including 57.7% women. We identified 29 participants with parkinsonism. The mean age at parkinsonism diagnosis was 72.6 (8.1) years and 52% of people with parkinsonism were women.

Code PRIME-NL study

L.J. Dommershuijsen

30/06/2021

Load packages

```
library("tidyverse")
library("car")
library("haven")
library("foreign")
library("tableone")
library("corrplot")
library("splines")
library("cowplot")
library("boot")
```

Load datasets

```
D <- read.csv2("P1_Algemene gegevens_corrected_2021-03-08_Version2.csv")
D <- as.data.frame(D)
Motor <- read.csv2("P8_MDS_UPDRS_processed_total_2021-03-08.csv")
Motor <- as.data.frame(Motor)
Non_Motor <- read.csv2("P9_Scopa_aut_processed_total_2021-03-08.csv")
Non_Motor <- as.data.frame(Non_Motor)
COVID <- read.csv2("P14.2_COVID_processed_total_2021-03-08.csv")
COVID <- as.data.frame(COVID)
BDI<- read.csv2("P7_BDI_processed_total_2021-03-08.csv")
BDI <- as.data.frame(BDI)
Anxiety <- read.csv2("P6_StaI_processed_total_2021-03-08.csv")
Anxiety <- as.data.frame(Anxiety)
QOL <- read.csv2("P2_pdq39_processed_total_2021-03-08.csv")
QOL <- as.data.frame(QOL)
MOCA <- read.csv2("Pt_StudyData_2021-03-08.csv")
colnames(MOCA)[1] <- "Castor.Record.ID"
MOCA <- dplyr::filter(MOCA, grepl("PT",Castor.Record.ID))
MOCA <- MOCA %>% filter(St_moca >=0)
Coping <- read.csv2("P3_wcq_processed_total_2021-03-08.csv")
Coping <- as.data.frame(Coping)
```

Clean dataset

```
## General covariates
D$primedate <- as.Date(D$Survey.Completed.On,tryFormats = c("%d-%m-%Y"))
D$yearnew <- as.numeric(ifelse((nchar(D$yearnew) > 4 | nchar(D$yearnew) < 4) & !is.na(D$yearnew), paste0("19", st
r_sub(D$yearnew, start= -2)), D$yearnew))
D$age <- (as.numeric(format(as.Date(D$primedate, format="%d-%m-%Y"),"%Y")) - D$yearnew)
D$age_group <- ifelse(D$age >= median(D$age, na.rm=T), 1, 0)
D$fup <- as.numeric(D$primedate - as.Date(paste("27-02-2020"),"%d-%m-%y"))/7
D$alg_gend <- as.numeric(D$alg_gend)-1 # men 0 women 1
D$region <- as.numeric(D[,'region'])-1  # 0=PRIME region; 1=Usual Care region
D <- D %>% mutate(alg_wn = case_when(alg_wn == 1 ~ 0, # Alone
                                     alg_wn == 2 | alg_wn == 3 ~ 1, # Together
                                     alg_wn == 4 | alg_wn == 5 | alg_wn == 6 ~ 2 # assisted living
))
```

```
D$alg_wn_tot <- D$alg_wn
D <- D %>% mutate(alg_wn = case_when(alg_wn == 0 ~ 0, # Alone
                                     alg_wn == 1 ~ 1)) # Together

D <- D %>% mutate(alg_ond = case_when(alg_ond == 1 | alg_ond == 2 ~ 0, # Primary
                                      alg_ond == 3 | alg_ond == 4 | alg_ond == 5 ~ 1, # Lower
                                      alg_ond == 6 | alg_ond == 7 ~ 2, # Intermediate
                                      alg_ond == 8 | alg_ond == 9 ~ 3 # Higher
))
```

```
D <- D %>% mutate(alg_ond_cat = case_when(alg_ond == 0 | alg_ond == 1 ~ 0,
                                          alg_ond == 2 | alg_ond == 3 ~ 1
))

D <- D %>% mutate(wrk = if_else(alg_wrk.Fulltime_dienstverband == 1 | alg_wrk.Parttime_dienstverband == 1 |  alg_
wrk.Zelfstandig == 1,  1,
                                if_else(alg_wrk.Gepensioneerd  == 1 | alg_wrk.Actief_in_het_huishouden_voor_kinde
ren_of_andere_pe0 == 1 | alg_wrk.Vrijwilligerswerk == 1 , 2,
                                        if_else(alg_wrk.Arbeidsongeschikt == 1 | alg_wrk.In_de_ziektewet == 1, 3,
                                                if_else(alg_wrk.Werkloos == 1, 2, NA_real_)))))
```

```
# Morbidity
D <- D %>% rename(hart_vaat = alg_aand.Hart_of_vaataandoening_Bijvoorbeeld_angina_pectori0,
                  long = alg_aand.Longaandoening_Bijvoorbeeld_astma_COPD_pulmonale_h0,
                  bewegingsapparaat = alg_aand.Aandoening_van_het_bewegingsapparaat_Hier_vallen_o0,
                  psychiatrie = alg_aand.Neuropsychiatrische_aandoening_Bijvoorbeeld_angsts0,
                  endocrien = alg_aand.Endocriene_of_metabole_aandoening_Bijvoorbeeld_dia0,
                  kanker = alg_aand.Kanker_Bijvoorbeeld_borst__darm_long_of_prostaatka0)

D$comorb <- ifelse(D$hart_vaat == 1 | D$long == 1 | D$bewegingsapparaat == 1 | D$psychiatrie == 1 | D$endocrien =
= 1 | D$kanker == 1, 1, 0)
D$psychiatrie <- ifelse(D$alg_psy_spec.Dementie == 1 & D$alg_psy_spec.Angststoornis != 1 & D$alg_psy_spec.Depress
ie != 1 & D$alg_psy_spec.Verslaving != 1, 0, D$psychiatrie)
D$alg_park_lft <- D$alg_park_lftnew
D$PD_duur <- D$age - D$alg_park_lft
D <- D %>% mutate(PD_duur_cat = case_when(PD_duur <=median(PD_duur, na.rm = T) ~ 0,
                                          PD_duur > median(PD_duur, na.rm = T) ~ 1))

# MDS UPDRS-II
Motor <- Motor[,c("Castor.Record.ID","MDS_UPDRS_Card_sum")]
D <- merge(D, Motor, all.x = T)
D <- D %>% mutate(motor_cat = case_when(MDS_UPDRS_Card_sum <= median(MDS_UPDRS_Card_sum, na.rm = T) ~ 0,
                                        MDS_UPDRS_Card_sum > median(MDS_UPDRS_Card_sum, na.rm = T) ~ 1))

# SCOPA-AUT
Non_Motor$scopa_aut_sum <- round((Non_Motor$scopa_aut_sum/63)*69)
D <- merge(D, Non_Motor[c("Castor.Record.ID","scopa_aut_sum")], by = "Castor.Record.ID", all.x = T)
D <- D %>% mutate(nonmotor_cat = case_when(scopa_aut_sum <= median(scopa_aut_sum, na.rm = T) ~ 0,
                                           scopa_aut_sum > median(scopa_aut_sum, na.rm = T) ~ 1))

# Cognition
MOCA <- MOCA[c("Castor.Record.ID","St_moca_mod","St_moca_complete")]
D <- merge(D, MOCA, by = "Castor.Record.ID", all.x = T)
D$MoCa <- ifelse(D$St_moca_complete == 0, NA, D$St_moca_mod)
D$MoCa_cat <- ifelse(D$MoCa >= median(D$MoCa, na.rm = T), 1, 0)
```

```
# Coping (Cases without stress were coded as NA)
Coping <- Coping[,c("Castor.Record.ID","WQCPDrel_confrontcoping", "WQCPDrel_distancing", "WQCPDrel_selfcontrol",
                    "WQCPDrel_seekingsocsupport","WQCPDrel_acceptrespons", "WQCPDrel_escapeavoidance",
                    "WQCPDrel_planfulprobsolving","WQCPDrel_posreappr" ),drop=FALSE]
D <- merge(D, Coping, by = "Castor.Record.ID", all.x = T)

D <- D %>% mutate(WQCPDrel_confrontcoping_cat = case_when(WQCPDrel_confrontcoping <= round(median(WQCPDrel_confro
ntcoping, na.rm = T)) ~ 0,
                                                          WQCPDrel_confrontcoping > round(median(WQCPDrel_confron
tcoping, na.rm = T)) ~ 1),
                  WQCPDrel_distancing_cat = case_when(WQCPDrel_distancing <= round(median(WQCPDrel_distancing, na
.rm = T)) ~ 0,
                                                      WQCPDrel_distancing > round(median(WQCPDrel_distancing, na.
rm = T)) ~ 1),
                  WQCPDrel_selfcontrol_cat = case_when(WQCPDrel_selfcontrol <= round(median(WQCPDrel_selfcontrol,
na.rm = T)) ~ 0,
                                                       WQCPDrel_selfcontrol > round(median(WQCPDrel_selfcontrol,
na.rm = T)) ~ 1),
                  WQCPDrel_seekingsocsupport_cat = case_when(WQCPDrel_seekingsocsupport <= round(median(WQCPDrel_
seekingsocsupport, na.rm = T)) ~ 0,
                                                             WQCPDrel_seekingsocsupport > round(median(WQCPDrel_s
eekingsocsupport, na.rm = T)) ~ 1),
                  WQCPDrel_acceptrespons_cat = case_when(WQCPDrel_acceptrespons <= round(median(WQCPDrel_acceptre
spons, na.rm = T)) ~ 0,
                                                         WQCPDrel_acceptrespons > round(median(WQCPDrel_acceptres
pons, na.rm = T)) ~ 1),
                  WQCPDrel_escapeavoidance_cat = case_when(WQCPDrel_escapeavoidance <= round(median(WQCPDrel_esca
peavoidance, na.rm = T)) ~ 0,
                                                           WQCPDrel_escapeavoidance > round(median(WQCPDrel_escap
eavoidance, na.rm = T)) ~ 1),
                  WQCPDrel_planfulprobsolving_cat = case_when(WQCPDrel_planfulprobsolving <= round(median(WQCPDre
l_planfulprobsolving, na.rm = T)) ~ 0,
                                                              WQCPDrel_planfulprobsolving > round(median(WQCPDrel
_planfulprobsolving, na.rm = T)) ~ 1),
                  WQCPDrel_posreappr_cat = case_when(WQCPDrel_posreappr <= round(median(WQCPDrel_posreappr, na.rm
= T)) ~ 0,
                                                     WQCPDrel_posreappr > round(median(WQCPDrel_posreappr, na.rm
= T)) ~ 1))

# COVID questionnaire
D <- merge(D, COVID, by = "Castor.Record.ID", all = T)
D$access_care <- rowSums(dplyr::select(D, CovPT_2:CovPT_4))/rowSums(!is.na(dplyr::select(D, CovPT_2:CovPT_4)))*3
D$social_influence <- rowSums(dplyr::select(D, CovPT_5,CovPT_6, CovPT_8))/rowSums(!is.na(dplyr::select(D, CovPT_5
,CovPT_6, CovPT_8)))*3
D$COV_total <- rowSums(dplyr::select(D, CovPT_1:CovPT_8))/(rowSums(!is.na(dplyr::select(D, CovPT_1:CovPT_8))))*8
```

```
# BDI
BDI <- BDI[c(6:28, 1)]
BDI[,'Bdi2It16']<- BDI$Bdi2It16mod
BDI[,'Bdi2It18']<- BDI$Bdi2It18mod
BDI$Bdi2It16mod <- NULL
BDI$Bdi2It18mod <- NULL
BDI$BDI_sum <- rowSums(BDI[,-ncol(BDI)])
BDI$cogn_affect <- rowSums(BDI[,c(1:14)])
BDI$somatic <- rowSums(BDI[,c(15:21)])
D <- merge(D, BDI, by = "Castor.Record.ID", all.x = T)

# STAI
Anxiety <- Anxiety %>% dplyr::select(Castor.Record.ID, StaiState01:StaiTrait40) %>% mutate_at(vars(-Castor.Record
.ID), as.numeric)
Anxiety <- Anxiety %>% mutate_at(vars(matches("01|02|05|08|10|11|15|16|19|20|21|23|26|27|30|33|34|36|39")),
                                 list(~case_when(. == 1 ~ 4,
                                                 . == 2 ~ 3,
                                                 . == 3 ~ 2,
                                                 . == 4 ~ 1))) # Reverse scores for anxiety-absent items
Anxiety <- Anxiety %>%
  mutate(nvalidState = rowSums(!is.na(dplyr::select(., contains("State")))),
         nvalidTrait = rowSums(!is.na(dplyr::select(., contains("Trait")))))

Anxiety <- Anxiety %>%
  mutate(StaiState_weighted = if_else(nvalidState > 17, round((rowSums(dplyr::select(., contains("State"), - nval
idState), na.rm = T)/nvalidState*20), 0), NA_real_)) %>%
  mutate(StaiTrait_weighted = if_else(nvalidTrait > 17, round((rowSums(dplyr::select(., contains("Trait"), - nval
idTrait), na.rm = T)/nvalidState*20), 0), NA_real_))

Anxiety <- Anxiety %>% mutate(StaiTotal_weighted = StaiState_weighted + StaiTrait_weighted)
D <- merge(D, Anxiety, by = "Castor.Record.ID", all.x = T)

# QOL
QOL <- QOL %>% dplyr::select(Castor.Record.ID, pdq39_mobility:average_pdq39)
QOL <- QOL %>% rename(QOL_Mobility = pdq39_mobility, QOL_ADL = pdq39_adl, QOL_Emotion = pdq39_emotional, QOL_Stig
ma = pdq39_stigma, QOL_Socialsupport = pdq39_support,
                      QOL_Cognition = pdq39_cognition, QOL_Communication = pdq39_communication, QOL_Bodily_discom
fort = pdq39_discomfort, QOL_sum = average_pdq39)
D <- merge(D, QOL, by = "Castor.Record.ID", all.x = T)
```

```
# Scale outcomes
D <- D %>% mutate(BDI_sum_orig = BDI_sum,
                  StaiTotal_weighted_orig = StaiTotal_weighted,
                  QOL_sum_orig = QOL_sum,
                  QOL_Mobility_orig = QOL_Mobility,
                  QOL_ADL_orig = QOL_ADL,
                  QOL_Emotion_orig = QOL_Emotion,
                  QOL_Stigma_orig = QOL_Stigma,
                  QOL_Socialsupport_orig = QOL_Socialsupport,
                  QOL_Cognition_orig = QOL_Cognition,
                  QOL_Communication_orig = QOL_Communication,
                  QOL_Bodily_discomfort_orig = QOL_Bodily_discomfort)

D <- D %>%
  mutate_at(c("StaiTotal_weighted", "StaiState_weighted", "StaiTrait_weighted", "BDI_sum", "cogn_affect", "somati
c", "QOL_sum", "QOL_Mobility", "QOL_ADL", "QOL_Emotion", "QOL_Stigma", "QOL_Socialsupport", "QOL_Cognition","QOL_
Communication", "QOL_Bodily_discomfort"), list(~ c(scale(.))))

# Keep only participants with PD
D <- subset(D, alg_diagn == 1)
```

Baseline table

```
# Include only those after April (start COVID questionnaire)
D <- subset(D, primedate > as.Date("2020-04-10")) # 851
# Include only those with information on COVID-19 questionnaire
D <- D[complete.cases(D$COV_total),] # 844

baselinetab_patients <-CreateTableOne(vars=c("age","alg_gend","region","alg_wn_tot","alg_ond","alg_etn.Nederlands
e", "wrk", "alg_diagn","alg_park_lft","PD_duur","MDS_UPDRS_Card_sum","scopa_aut_sum","comorb","MoCa",
                                              "hart_vaat","long","bewegingsapparaat","psychiatrie","endocrien","ka
nker",
                                              "CovPT_1","CovPT_2","CovPT_3", "CovPT_4","CovPT_5","CovPT_6","CovPT_
7","CovPT_8",
                                              "access_care","social_influence","COV_total",
                                              "QOL_sum_orig","StaiTotal_weighted_orig","BDI_sum_orig", "cogn_affec
t", "somatic", "StaiState_weighted", "StaiTrait_weighted"),
                                       data=D, factorVars = c("alg_gend","region","alg_wn_tot","alg_ond","alg_etn.
Nederlandse", "wrk", "alg_diagn","comorb",
                                                              "hart_vaat","long","bewegingsapparaat","psychiatrie"
,"endocrien","kanker")
)
baselinetab_patients <- as.data.frame(print(baselinetab_patients,  nonnormal = c("CovPT_1","CovPT_2","CovPT_3", "
CovPT_4","CovPT_5","CovPT_6","CovPT_7","CovPT_8"),quote = FALSE, noSpaces = TRUE, printToggle = FALSE))
```

Linear models

```
Outcomes <- as.data.frame(c("StaiTotal_weighted", "BDI_sum", "QOL_sum","StaiState_weighted", "StaiTrait_weighted"
,"cogn_affect", "somatic","QOL_Mobility","QOL_ADL","QOL_Emotion","QOL_Stigma","QOL_Socialsupport","QOL_Cognition"
,"QOL_Communication","QOL_Bodily_discomfort"))
colnames(Outcomes) <- "Outcomes"
Variables <- as.data.frame(c("CovPT_1",
                             "CovPT_2",
                             "CovPT_3",
                             "CovPT_4",
                             "CovPT_5",
                             "CovPT_6",
                             "CovPT_7",
                             "CovPT_8",
                             "access_care",
                             "social_influence",
                             "COV_total"))

colnames(Variables) <- "Variables"
Stratification <- c("",
                    "alg_gend",
                    "age_group",
                    "alg_ond_cat",
                    "comorb",
                    "psychiatrie",
                    "alg_wn",
                    "PD_duur_cat",
                    "region",
                    "motor_cat",
                    "nonmotor_cat",
                    "MoCa_cat",
                    "WQCPDrel_confrontcoping_cat",
                    "WQCPDrel_distancing_cat",
                    "WQCPDrel_selfcontrol_cat",
                    "WQCPDrel_seekingsocsupport_cat",
                    "WQCPDrel_acceptrespons_cat",
                    "WQCPDrel_escapeavoidance_cat",
                    "WQCPDrel_planfulprobsolving_cat",
                    "WQCPDrel_posreappr_cat")

full <- "+ alg_gend + age + PD_duur + comorb + alg_ond + alg_wn_tot + region + fup"
Adjustment <- as.data.frame(c("+ alg_gend + age+ fup",
                              "+ age + PD_duur + comorb + alg_ond + alg_wn_tot + region+ fup",
                              "+ alg_gend + PD_duur + comorb + alg_ond + alg_wn_tot + region+ fup",
                              "+ alg_gend + age + PD_duur + comorb + alg_wn_tot + region+ fup",
                              "+ alg_gend + age + PD_duur + alg_ond + alg_wn_tot + region+ fup",
                              full,
                              "+ alg_gend + age + PD_duur + comorb + alg_ond + region+ fup",
                              "+ alg_gend + age + comorb + alg_ond + alg_wn_tot + region+ fup",
                              "+ alg_gend + age + PD_duur + comorb + alg_ond + alg_wn_tot+ fup",
                              rep(full, 11)))

Output <- list()

count <- 1
```

```r
for (m in 1:length(Stratification)){
  for (d in 1:nrow(Variables)){
    for (g in 1:nrow(Outcomes)){
      if(m == 1){
        Output[[count]] <- with(D, lm(as.formula(paste0(Outcomes[g,1], "~", Variables[d,1], Adjustment[m,1]))))
        names(Output)[count] <- paste0(Outcomes[g,1], ":", Variables[d,1], ":","NA", ":", "NA", ":","model1")
        count <- count+1
        Output[[count]] <- with(D, lm(as.formula(paste0(Outcomes[g,1], "~", Variables[d,1], Adjustment[(m+5),1]))
))
        names(Output)[count] <- paste0(Outcomes[g,1], ":", Variables[d,1], ":", "NA", ":", "NA", ":","model2")
        count <- count+1
      }
      if(nlevels(factor(D[[Stratification[m]]])) == 2 & (d == 9 | d == 10 | d == 11) & (g == 1 | g == 2 |  g == 3
)){
        Output[[count]] <- with(D[D[[Stratification[m]]] == 0,], lm(as.formula(paste0(Outcomes[g,1], "~", Variabl
es[d,1], Adjustment[m,1]))))
        names(Output)[count] <- paste0(Outcomes[g,1], ":", Variables[d,1], ":",Stratification[m], ":","0",":","mo
del2")
        count <- count+1
        Output[[count]] <- with(D[D[[Stratification[m]]] == 1,], lm(as.formula(paste0(Outcomes[g,1], "~", Variabl
es[d,1], Adjustment[m,1]))))
        names(Output)[count] <- paste0(Outcomes[g,1], ":", Variables[d,1], ":",Stratification[m], ":","1",":","mo
del2")
        count <- count+1
      }
    }
  }
}

# check assumptions
# vif(Output[[50]])
# for(i in 1:length(Output)){
#   par(mfrow = c(2, 2))
#   p<- plot(Output[[i]], main = names(Output)[i])
#   print(p)
# }

res <- lapply(seq_along(Output), function(i) {
  data.frame(determinants = names(Output)[i],
             coef = Output[[i]]$coefficients[2],
             confint_low = confint(Output[[i]])[2,1],
             confint_high = confint(Output[[i]])[2,2],
             p_value = summary(Output[[i]])$coefficients[2,4])
})

output_df <- do.call(rbind, res)
rm(list=setdiff(ls(), c("output_df", "D")))
par(mfrow=c(1,1))
```

Preparation for plots

```r
# Code for creation of plots not shown
```

Hypothetical interventions

```r
D <- D %>% filter(!is.na(QOL_sum_orig)) %>% filter(!is.na(COV_total)) %>% filter(!is.na(BDI_sum_orig)) %>% filter
(!is.na(StaiTotal_weighted_orig)) %>% filter(!is.na(alg_gend)) %>% filter(!is.na(age)) %>% filter(!is.na(PD_duur)
) %>% filter(!is.na(comorb)) %>% filter(!is.na(alg_ond)) %>% filter(!is.na(alg_wn_tot)) %>% filter(!is.na(region)
)

# Function to calculate difference in means
standardization <- function(data, indices, outcome, determinant, adjustment, adjustment2) {
  # create a dataset with X copies of each subject
  d <- data[indices, ] # 1st copy: equal to original one`
  d$interv <- 0
  d$riskgroup1 <- ifelse(d[,determinant] > median(d[,determinant]), 1, 0)
  d$riskgroup2 <- ifelse(d$motor_cat == 1, 1, 0)

  d1 <- d # 2nd copy: intervention set to 1, outcome to missing, determinant to 0 (100% reduction)
  d1$interv <- 1
  d1[,determinant] <- 0
  d1[,outcome] <- NA

  d2 <- d # 3rd copy: intervention set to 2, outcome to missing, determinant to half (50% reduction)
  d2$interv <- 2
  d2[,determinant] <- round(d2[,determinant]/2)
  d2[,outcome] <- NA
```

```
  d3 <- d # 3rd copy: intervention set to 3, outcome to missing, determinant to 3/4 (25% reduction)
  d3$interv <- 3
  d3[,determinant] <- round(d3[,determinant]/4*3)
  d3[,outcome] <- NA

  d4 <- d # 4th copy: intervention set to 4, in risk group: outcome to missing, determinant to half (50% reductio
n)
  d4$interv <- 4
  d4[,determinant] <- round(ifelse(d4$riskgroup1 == 1, d4[,determinant]/2,d4[,determinant]),0)
  d4[,outcome] <- NA

  d5 <- d # 4th copy: intervention set to 5, in risk group: outcome to missing, determinant to 3/4 (25% reduction
)
  d5$interv <- 5
  d5[,determinant] <- round(ifelse(d5$riskgroup1 == 1, d5[,determinant]/4*3,d5[,determinant]),0)
  d5[,outcome] <- NA

  d6 <- d # 5th copy: intervention set to 6, in risk group: outcome to missing, determinant to half (50% reductio
n)
  d6$interv <- 6
  d6[,determinant] <- ifelse(d6$riskgroup2 == 1, d6[,determinant]/2, d6[,determinant])
  d6[,outcome] <- NA

  d7 <- d # 5th copy: intervention set to 7, in risk group: outcome to missing, determinant to half (50% reductio
n)
  d7$interv <- 7
  d7[,determinant] <- ifelse(d7$riskgroup2 == 1, d7[,determinant]/4*3, d7[,determinant])
  d7[,outcome] <- NA

  d.onesample <- rbind(d, d1, d2, d3, d4, d5, d6, d7)

  d.onesample$predicted_meanY <- ifelse(d.onesample$interv != 5 & d.onesample$interv != 6, predict(lm(
    as.formula(paste0(outcome, "~", determinant, adjustment)), data = d.onesample
  ), d.onesample), predict(lm(
    as.formula(paste0(outcome, "~", determinant, adjustment2)), data = d.onesample
  ), d.onesample))

  # estimate mean outcome in each of the groups
  return(c(
    mean(d.onesample$predicted_meanY[d.onesample$interv == 0]),
    mean(d.onesample$predicted_meanY[d.onesample$interv == 1]),
    mean(d.onesample$predicted_meanY[d.onesample$interv == 2]),
    mean(d.onesample$predicted_meanY[d.onesample$interv == 3]),
    mean(d.onesample$predicted_meanY[d.onesample$interv == 4]),
    mean(d.onesample$predicted_meanY[d.onesample$interv == 5]),
    mean(d.onesample$predicted_meanY[d.onesample$interv == 6]),
    mean(d.onesample$predicted_meanY[d.onesample$interv == 7]),

    # mean for at riskgroup1 only
    mean(d.onesample[d.onesample$interv == 0 & d.onesample$riskgroup1 == 1, outcome]),
    mean(d.onesample$predicted_meanY[d.onesample$interv == 4 & d.onesample$riskgroup1 == 1]),
    nrow(subset(d.onesample, interv == 4 & riskgroup1 == 1)),
    mean(d.onesample$predicted_meanY[d.onesample$interv == 5 & d.onesample$riskgroup1 == 1]),

    # mean for at riskgroup2 only
    mean(d.onesample[d.onesample$interv == 0 & d.onesample$riskgroup2 == 1, outcome]),
    mean(d.onesample$predicted_meanY[d.onesample$interv == 6 & d.onesample$riskgroup2 == 1]),
    nrow(subset(d.onesample, interv == 6 & riskgroup2 == 1)),
    mean(d.onesample$predicted_meanY[d.onesample$interv == 7 & d.onesample$riskgroup2 == 1])
  ))
}


# generating confidence intervals
bootstrap_CI <- function(results) {
  se <- c(sd(results$t[, 1]),
          sd(results$t[, 2]))
  mean <- results$t0
  ll <- mean - qnorm(0.975) * se
  ul <- mean + qnorm(0.975) * se
  data.frame(cbind(
    c(
      "Observed", # Creates row for observed
      "Treatment1", # Creates row for prediction after treatment
      "Treatment2", # Creates row for prediction after treatment
      "Treatment3", # Creates row for prediction after treatment
      "Treatment4", # Creates row for prediction after treatment
      "Treatment5", # Creates row for prediction after treatment
      "Treatment6", # Creates row for prediction after treatment
```

```
      "Treatment7", # Creates row for prediction after treatment
      "Treatment4_orig", # Creates row for original outcome treated
      "Treatment4_treated", # Creates row for treated only
      "N intervention 4", # Creates row for number treated
      "Treatment5_treated", # Creates row for treated only
      "Treatment6_orig", # Creates row for original outcome treated
      "Treatment6_treated", # Creates row for treated only
      "N intervention 6", # Creates row for number treated
      "Treatment7_treated" # Creates row for treated only
    ),
    mean,
    se,
    ll,
    ul
  ))
}

# BDI social
set.seed(2021)
BDI_social_results <- boot(data = D, outcome = "BDI_sum_orig", determinant = "social_influence", adjustment = "+
alg_gend + age + PD_duur + comorb + alg_ond + alg_wn_tot + region + fup", adjustment2 = "+ alg_gend + age + comor
b + alg_ond + alg_wn_tot + region + fup",
                           statistic = standardization,
                           R = 1000)
BDI_social_results <- bootstrap_CI(BDI_social_results)
# BDI care
set.seed(2021)
BDI_care_results <- boot(data = D, outcome = "BDI_sum_orig", determinant = "access_care", adjustment = "+ alg_gen
d + age + PD_duur + comorb + alg_ond + alg_wn_tot + region + fup", adjustment2 = "+ alg_gend + age + comorb + alg
_ond + alg_wn_tot + region + fup",
                         statistic = standardization,
                         R = 1000)
BDI_care_results <- bootstrap_CI(BDI_care_results)
# BDI total
set.seed(2021)
BDI_total_results <- boot(data = D, outcome = "BDI_sum_orig", determinant = "COV_total", adjustment = "+ alg_gend
+ age + PD_duur + comorb + alg_ond + alg_wn_tot + region + fup", adjustment2 = "+ alg_gend + age + comorb + alg_o
nd + alg_wn_tot + region + fup",
                          statistic = standardization,
                          R = 1000)
BDI_total_results <- bootstrap_CI(BDI_total_results)

# STAI sociaL
set.seed(2021)
STAI_social_results <- boot(data = D, outcome = "StaiTotal_weighted_orig", determinant = "social_influence", adju
stment = "+ alg_gend + age + PD_duur + comorb + alg_ond + alg_wn_tot + region + fup", adjustment2 = "+ alg_gend +
age + comorb + alg_ond + alg_wn_tot + region + fup",
                            statistic = standardization,
                            R = 1000)
STAI_social_results <- bootstrap_CI(STAI_social_results)
# STAI care
set.seed(2021)
STAI_care_results <- boot(data = D, outcome = "StaiTotal_weighted_orig", determinant = "access_care", adjustment
= "+ alg_gend + age + PD_duur + comorb + alg_ond + alg_wn_tot + region + fup", adjustment2 = "+ alg_gend + age +
comorb + alg_ond + alg_wn_tot + region + fup",
                          statistic = standardization,
                          R = 1000)
STAI_care_results <- bootstrap_CI(STAI_care_results)
# STAI total
set.seed(2021)
STAI_total_results <- boot(data = D, outcome = "StaiTotal_weighted_orig", determinant = "COV_total", adjustment =
"+ alg_gend + age + PD_duur + comorb + alg_ond + alg_wn_tot + region + fup", adjustment2 = "+ alg_gend + age + co
morb + alg_ond + alg_wn_tot + region + fup",
                           statistic = standardization,
                           R = 1000)
STAI_total_results <- bootstrap_CI(STAI_total_results)

# QOL sociaL
set.seed(2021)
QOL_social_results <- boot(data = D, outcome = "QOL_sum_orig", determinant = "social_influence", adjustment = "+
alg_gend + age + PD_duur + comorb + alg_ond + alg_wn_tot + region + fup", adjustment2 = "+ alg_gend + age + comor
b + alg_ond + alg_wn_tot + region + fup",
                           statistic = standardization,
                           R = 1000)
QOL_social_results <- bootstrap_CI(QOL_social_results)
# QOL care
set.seed(2021)
QOL_care_results <- boot(data = D, outcome = "QOL_sum_orig", determinant = "access_care", adjustment = "+ alg_gen
d + age + PD_duur + comorb + alg_ond + alg_wn_tot + region + fup", adjustment2 = "+ alg_gend + age + comorb + alg
```

```
_ond + alg_wn_tot + region + fup",
                         statistic = standardization,
                         R = 1000)
QOL_care_results <- bootstrap_CI(QOL_care_results)
# QOL total
set.seed(2021)
QOL_total_results <- boot(data = D, outcome = "QOL_sum_orig", determinant = "COV_total", adjustment = "+ alg_gend
+ age + PD_duur + comorb + alg_ond + alg_wn_tot + region + fup", adjustment2 = "+ alg_gend + age + comorb + alg_o
nd + alg_wn_tot + region + fup",
                          statistic = standardization,
                          R = 1000)
QOL_total_results <- bootstrap_CI(QOL_total_results)

# Table outcomes
Outcomes <- rbind(BDI_total_results, BDI_social_results[c(2:8),], BDI_care_results[c(2:8),], STAI_total_results,
STAI_social_results[c(2:8),], STAI_care_results[c(2:8),], QOL_total_results, QOL_social_results[c(2:8),], QOL_car
e_results[c(2:8),])

Outcomes$se <- NULL
Outcomes <- Outcomes %>% mutate_at(vars(mean, ll, ul), as.character)
Outcomes <- Outcomes %>% mutate_at(vars(mean, ll, ul), as.numeric)
Outcomes[,2:4] <- round(Outcomes[,2:4],1)
```
